# Effect of the Flavonoid Rutin on the Modulation of the Myenteric Plexuses in an Experimental Model of Parkinson’s Disease

**DOI:** 10.3390/ijms25021037

**Published:** 2024-01-15

**Authors:** Livia Bacelar de Jesus, Annyta Fernandes Frota, Fillipe Mendes de Araújo, Rafael Leonne Cruz de Jesus, Maria de Fátima Dias Costa, Darizy Flavia Silva Amorim de Vasconcelos, Marcelo Biondaro Gois, Gyselle Chrystina Baccan, Victor Diogenes Amaral da Silva, Silvia Lima Costa

**Affiliations:** 1 Laboratory of Neurochemistry and Cellular Biology, Federal University of Bahia, Salvador 40170-110, BA, Brazil; liviabacelar@hotmail.com (L.B.d.J.); annyta.frota@gmail.com (A.F.F.); fillipemendesbio@gmail.com (F.M.d.A.); fatima@ufba.br (M.d.F.D.C.); 2 Cardiovascular Physiology and Pharmacology Laboratory, Federal University of Bahia, Salvador 40170-110, BA, Brazil; rafaelleonne@gmail.com (R.L.C.d.J.); darizy.silva@ufba.br (D.F.S.A.d.V.); 3 National Institute for Translational Neurosciences (INCT/CNPq INNT), Rio de Janeiro 21941-902, RJ, Brazil; 4 Faculty of Health Sciences, Federal University of Rondonópolis, Rondonópolis 78736-900, MT, Brazil; marcelobiondaro@gmail.com; 5 Laboratory of Neuroendocrine-Immunology, Federal University of Bahia, Salvador 40170-110, BA, Brazil; gbaccan@ufba.br

**Keywords:** rutin, enteric nervous system, Parkinson’s disease

## Abstract

Recent discoveries have shown that enteric glial cells play an important role in different neurodegenerative disorders, such as Parkinson’s disease (PD), which is characterized by motor dysfunctions caused by the progressive loss of dopaminergic neurons in the substance nigra pars compacta and non-motor symptoms including gastrointestinal dysfunction. In this study, we investigated the modulatory effects of the flavonoid rutin on the behavior and myenteric plexuses in a PD animal model and the response of enteric glia. Adult male Wistar rats were submitted to stereotaxic injection with 6-hydroxydopamine or saline, and they were untreated or treated with rutin (10 mg/kg) for 14 days. The ileum was collected to analyze tissue reactivity and immunohistochemistry for neurons (HuC/HuD) and enteric glial cells (S100β) in the myenteric plexuses. Behavioral tests demonstrated that treatment with rutin improved the motor capacity of parkinsonian animals and improved intestinal transit without interfering with the cell population; rutin treatment modulated the reactivity of the ileal musculature through muscarinic activation, reducing relaxation through the signaling pathway of nitric oxide donors, and increased the longitudinal contractility of the colon musculature in parkinsonian animals. Rutin revealed modulatory activities on the myenteric plexus, bringing relevant answers regarding the effect of the flavonoid in this system and the potential application of PD adjuvant treatment.

## 1. Introduction

The enteric nervous system (ENS) is a complex network of neurons and enteric glial cells organized into thousands of small ganglia, most of which are found in two plexuses: the submucosal plexus, found in the submucosa of the small and large intestine, and the myenteric plexus, located between the longitudinal and circular muscle layers, forming a continuous network extending from the upper part of the esophagus to the internal anal sphincter. The connections between the ENS and the central nervous system (CNS) are carried by the vagus and pelvic nerves and the sympathetic pathways [1,2]. The ENS coordinates important sensory and motor functions of the gastrointestinal tract (GIT), such as the delivery of digestive enzymes, the mixture and propagation of gastrointestinal contents, absorption, fluid exchange, storage and excretion, the secretion of enteric neuroendocrine and epithelial cells, immune response, the maintenance of intestinal epithelial barrier, and blood flow [1,2,3,4,5,6]. Studies have shown bidirectional interactions between the ENS and the CNS. Current knowledge about the gut–brain axis allows us to consider the relationship developed by these two systems in Parkinson’s disease (PD). This chronic, progressive, age-associated degenerative disease results in the loss of dopaminergic neurons in the substantia nigra and motor symptoms. In addition, gastrointestinal symptoms, such as constipation and abdominal pain, are observed in patients with PD [7,8,9,10,11].

Although significant advances in the understanding of the etiopathology of PD have been made, there are still few therapeutic approaches available. For this reason, there is a need to develop new adjuvant therapeutic strategies that prevent or delay the progression of the disease and that can act in the ENS, improving the gastric intestinal disorders observed in this pathology. Agents that operate as antioxidants and anti-inflammatories have been shown to be promising in PD [12,13,14]. In this context, flavonoids, which are compounds derived from the secondary metabolism of plants, have drawn attention in the scientific community. These compounds may prosecute neuroprotective effects on multiple pathological processes associated with neurodegenerative diseases [15]. Recent works have shown that flavonoids present antioxidant actions, modulate the inflammatory response, and are neuroprotective in neurodegenerative disorders [16,17]. Rutin (3,3′,4′,5,7–pentahydroxyflavone–3–rutinoside) is a glycosylated flavonoid that is present in many fruits and plants. Rutin is also obtained from the fruits of Dimorphandra mollis (Benth.) trees, a medicinal Brazilian plant that is an important source of this flavonoid. Studies have shown associative anti-inflammatory and antioxidant properties, resulting in the inhibition of nitric oxide (NO) production and the reduction in pro-inflammatory cytokines, and neuroprotective effects of purified flavonoid and plant extracts containing rutin [17,18,19]. Although very promising, there are still few studies that use flavonoids, especially rutin, as an agent that promotes the integrity and improvement of intestinal function in PD. In light of this, the present work aimed to investigate the flavonoid rutin in the behavior and myenteric plexus in an experimental animal model of PD, and also the response of enteric glia to the flavonoid.

## 2. Results

### 2.1. Rutin Treatment Reduced the Number of Contralateral Rotations Induced by 6-OHDA

**Figure 1:**
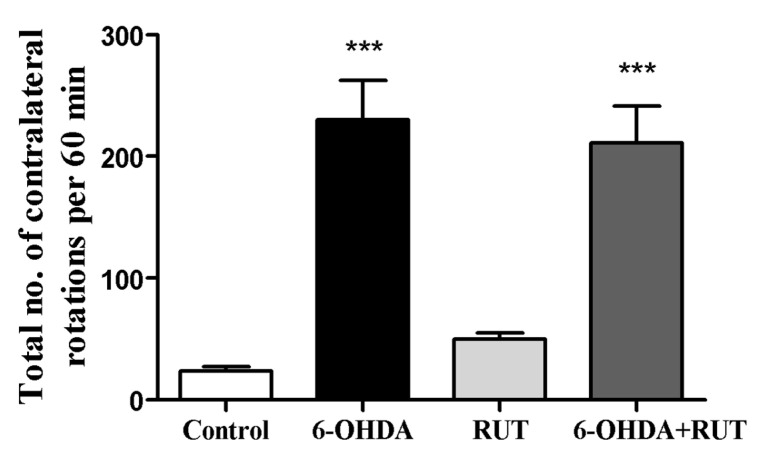
Analysis of the contralateral rotations induced by apomorphine in Wistar rats treated with 6-OHDA and/or rutin or in control conditions (not treated). The test was performed 14 days after intra-striatal injection with 6-OHDA. The values show mean ± S.E.M; *n* = 8 per group; *** indicates *p* < 0.001 in relation to the control group.

In this work, we analyzed the ability of the flavonoid rutin (10 mg/kg/day) to modulate ENS in a PD model induced by 6-hydroxydopamine (6-OHDA). The apomorphine-induced rotation test was performed to investigate the hypersensitivity of the lesioned striatum on the 14th day after stereotactic brain surgery. The evaluation of spontaneous rotation showed that the injured 6-OHDA animals spontaneously rotated contralaterally (230 ± 32.6) when compared with the control group. However, animals treated with rutin reduced spontaneous rotation significantly by 8% (211.4 ± 30.3) in comparison with the 6-OHDA-injured group (Figure 1). The animals that were treated only with rutin (49.7 ± 5.0) did not present a difference in this parameter in relation to the negative control group.

### 2.2. Rutin Treatment Improves the Motor Capacity of Animals

**Figure 2:**
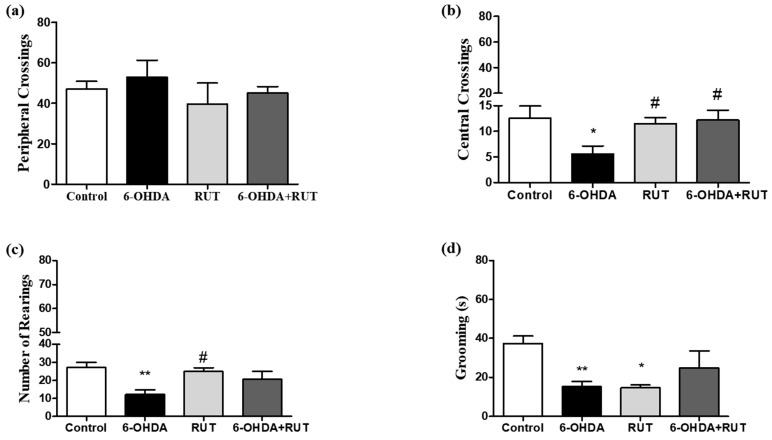
Behavioral damage in Wistar rats treated with 6-OHDA and/or rutin or in control conditions (not treated). Open field test (**a**–**d**). Peripheral crossings (**a**). Center crossings (**b**). Vertical counts (rearing) (**c**). Grooming (**d**). The values show mean ± S.E.M. * and ** indicate *p* < 0.05 and *p* < 0.01, respectively, in relation to the control group; *n* = 8 per group; # indicates *p* < 0.05 in relation to the 6-OHDA group.

The open-field test (Figure 2) was performed 14 days after the striatal injections. In the animals subjected to 6-OHDA damage (parkinsonian animals), changes were observed in the peripheral crossings (53 ± 8.3), the number of center crossings (5.6 ± 1.5), and number of rearings (12.2 ± 2.5) in the open-field test when compared with the control animals (47.1 ± 3.7, 12.6 ± 2.3, 27.1 ± 2.9, respectively). The animals that were treated with rutin presented a significant reduction in the parameter of peripheral crossings (45 ± 3.3), and presented increases in central crossings (12.2 ± 1.9) and rearings (20.7 ± 4.2). The animals that received only rutin presented similar results in relation to the control animals.

### 2.3. Rutin Reduces 6-OHDA-Induced Contralateral Deviation

**Figure 3:**
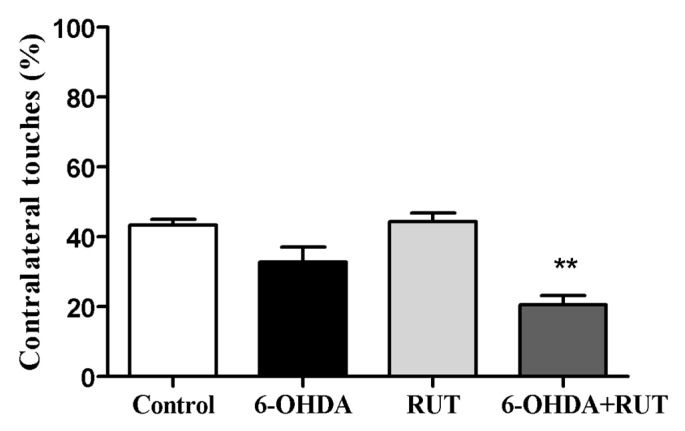
Rutin decreases the asymmetry of the forelimbs of parkinsonian animals, as assessed in the cylinder test. The test was performed on Wistar rats treated with 6-OHDA, rutin, 6-OHDA and rutin, or in control conditions (not treated) 14 days after intra-striatal injection with 6-OHDA. The percentages were calculated as the number of contralateral touches out of the total touches performed during the test. The values show mean ± S.E.M; *n* = 8 per group; ** indicates *p* < 0.01 in relation to the control group.

The 6-OHDA-treated animals showed a marked deficit in the use of the contralateral paw compared with the ipsilateral paw (touches with the contralateral paw account for 32.7 ± 4.3% of total touches). The animals that were co-treated with rutin showed an increase in the use of the contralateral paw compared with the ipsilateral paw (20.5 ± 2.7% of total touches). However, the animals that were treated only with rutin presented an increase in the use of the contralateral paw (44.2 ± 2.5) compared to the 6-OHDA-lesioned animals. As expected, the control animals did not present signs of behavioral impairment in the cylinder test (43.3 ± 1.7) (Figure 3).

### 2.4. Rutin Improves Gastrointestinal Transit and Increases Fecal Production in Parkinsonian Animals

**Figure 4:**
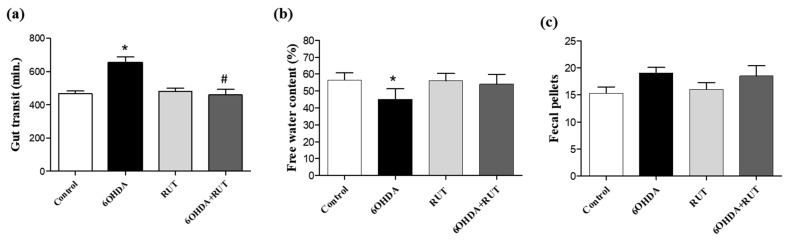
Intestinal transit was altered in parkinsonian rats. Whole gut transit test (**a**); free water content in the expelled fecal pelotons (**b**); number of fecal pellets produced by each group (**c**). The time taken to excrete the head of an orally administered marker (whole gut transit time) was measured. The 6-OHDA group demonstrated a longer gastrointestinal transit time and a lower moisture content in the feces formed. There was no difference in the number of fecal pellets formed between the groups (* *p* > 0.05, in relation to the control group; # indicates *p* < 0.05 in relation to the 6-OHDA group). One-Way ANOVA followed by Bonferroni post-test; *n* = 8 per group).

The parkinsonian animals showed impaired gastrointestinal transit, which was revealed by an increase in the gastric emptying time when compared with the control animals (200 min *p* < 0.05), and it was not observed in the animals that received treatment with rutin (10 mg/kg) (Figure 4a). In addition, a lower free water content was observed in the feces of animals in the 6-OHDA group when compared with the control animals (20%, *p* < 0.05) (Figure 4b). On the other hand, no significant differences were observed in the number of fecal pellets produced in the groups of experimental animals studied (Figure 4c).

### 2.5. Rutin Improves Reactivity to Muscarinic Receptors and Hinders Nitric Oxide Signaling in the Ileal Segments from Parkinsonian Animals

**Figure 5:**
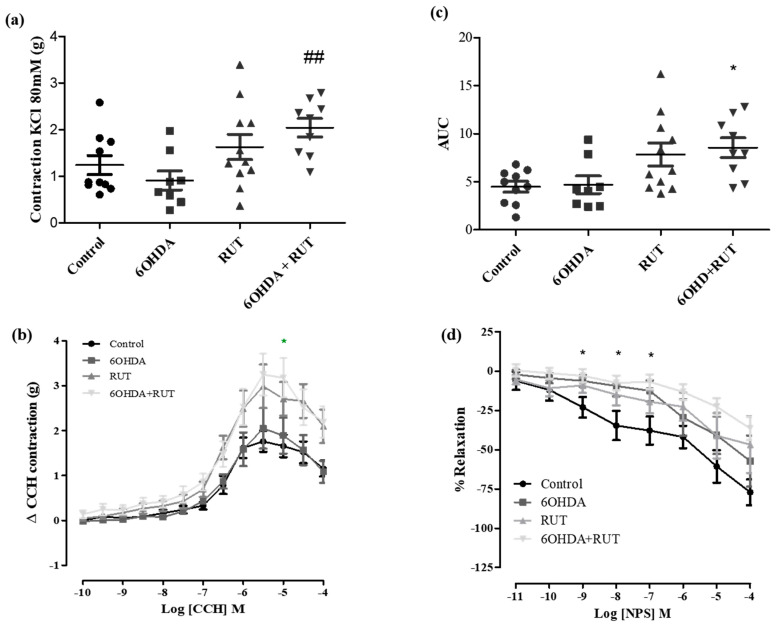
Effect of treatment with 6-OHDA and/or rutin in ileum reactivity. KCl induced contraction (80 mM) (g) (**a**); contraction variation in grams with cumulative concentrations of CCH (10^−10^–10^−4^) (**b**); bar graph with AUC values (area under the concentration–response curve for CCH) (**c**); percentage of relaxation in cumulative concentrations of NPS (10^−10^–10^−4^) (**d**). Control, 6-OHDA, rutin, and 6-OHDA+rutin group. Values are expressed as mean ± S.E.M; * *p* < 0.05 (6-OHDA+rutin vs. control) and ## *p* < 0.01 (6-OHDA+rutin vs. 6-OHDA). One-way ANOVA followed by Bonferroni post-test; *n* = 8 per group.

In order to determine the effects of rutin treatment on the intestinal smooth muscle contractility, we performed experiments using ileal segments stimulated with different contraction or relaxation factors. Figure 5a shows that the ileal contractions induced by the depolarizing solution (Tyrode’s solution containing KCl 80 mM) were greater in the rutin-treated 6-OHDA group than in the other groups. No significant difference was observed between the other groups, as shown in Figure 5.

In addition, carbachol-induced contraction was increased in ileal segments from parkinsonian animals treated with rutin compared with the control group (3.25 ± 0.46 vs. 1.65 ± 0.24 g/g of tissue, respectively; *p* < 0.05). Also, no significant difference was observed in the 6-OHDA and rutin groups when compared with the control.

On the other hand, NPS (a nitric oxide donor, 10^−11^–10^−4^ M) induced a relaxation effect of the ileal segments in a concentration-dependent manner. The NPS-induced relaxation was significantly reduced (Effect (10^−7^ M) = 6.8 ± 4.6%, *p* < 0.05) in the rutin-treated parkinsonian animals compared with the control group (Effect (10^−7^ M) = 37.9 ± 8.9% *p* < 0.05).

### 2.6. Treatment with Rutin Increases the Contractility of the Colonic Longitudinal Muscle from Parkinsonian Animals

**Figure 6:**
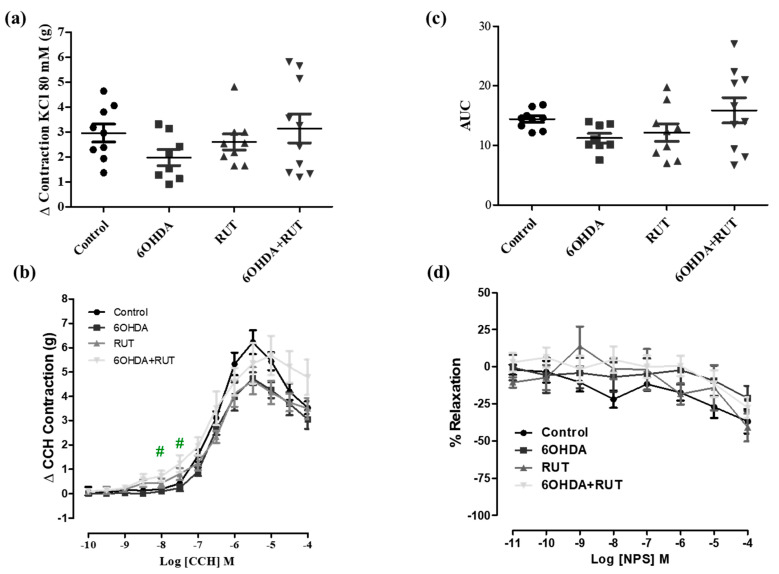
Effect of the treatment with 6-OHDA and/or rutin on colon reactivity. Contraction induced by 80 mM of KCl (g) (**a**); contraction variation in grams with cumulative concentrations of CCH (10^−10^–10^−4^) (**b**); bar graph with AUC value (area under the concentration–response curve for CCH) (**c**); percentage of relaxation in cumulative concentrations of NPS (10^−10^–10^−4^) (**d**). Control, 6-OHDA, rutin, and 6-OHDA+rutin group. Values are expressed as mean ± S.E.M. # *p* < 0.05 (6-OHDA+rutin vs. 6-OHDA). One-way ANOVA followed by Bonferroni post-test; *n* = 8 per group.

To assess whether treatment with rutin promoted changes in the contraction or relaxation of the colonic longitudinal muscle, concentration–response curves for 80 of KCl mM, carbachol, and NPS were performed. Figure 6 demonstrates that the effects induced by 80 mM of KCl and NPS were not changed among the experimental groups. However, carbachol-induced contraction was significantly increased in the colon segments from parkinsonian animals treated with rutin compared with the control group (Effect (3 × 10^−7^ M) = 0.23 ± 0.13 vs. 1.23 ± 0.34 g/g of tissue, respectively; *p* < 0.05).

### 2.7. Rutin Does Not Interfere in the Population of Glial Cells and Enteric Neurons of 6-OHDA-Injured Animals

**Figure 7:**
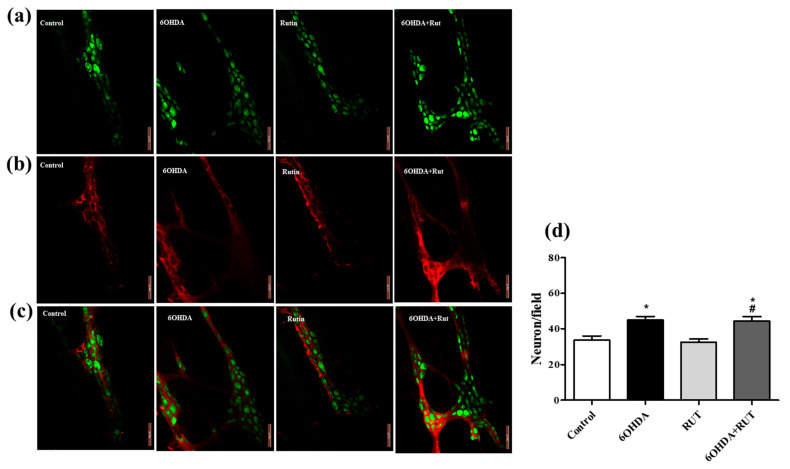
Photomicrographs of the myenteric plexus in the ileum of Wistar rats. Control, 6-OHDA, rutin group, and 6-OHDA+rutin group. (**a**) Green HuC/HuD neurons. (**b**) Red S100β glia. (**c**) Merge. An increase was observed in the number of neurons in the 6-OHDA and 6-OHDA+rutin groups (**d**). * *p* < 0.05 (6-OHDA+rutin vs. control) and # *p* < 0.05 (6-OHDA+rutin vs. 6-OHDA). One-way ANOVA was conducted, followed by Bonferroni post-test; *n* = 8 per group; scale bar = 100 μm.

The immunohistochemical staining for the neuron-specific HuC/HuD protein showed an increase of around 35% (p < 0.05) in myenteric plexus neurons in the ileum of animals with Parkinsonism that suffered damage caused by the nigrostrial injection of the neurotoxin 6-OHDA (Figure 7a–c). This increase was also observed in animals with Parkinsonism that were treated with rutin. On the other hand, no significant difference was observed in the number of myenteric plexus neurons in the ileum of animals treated with rutin alone compared with the control animals (Figure 7d). This effect shows that rutin, in this experimental model, does not interfere with the population of neurons in this region. A significant increase of approximately 47% (p < 0.5) was observed in the proportion of enteric glial cells (S100β positive) in the myenteric plexus of the ileum in parkinsonian animals that were untreated or treated with rutin when compared with the control animals. The treatment of animals with rutin alone also did not show a significant difference in the number of enteric glial cells compared with the control animals (Figure 7b).

### 2.8. Treatment with Rutin Protects from Damage Caused by IL-1β in Enteroglial Cells (EGCs)

**Figure 8:**
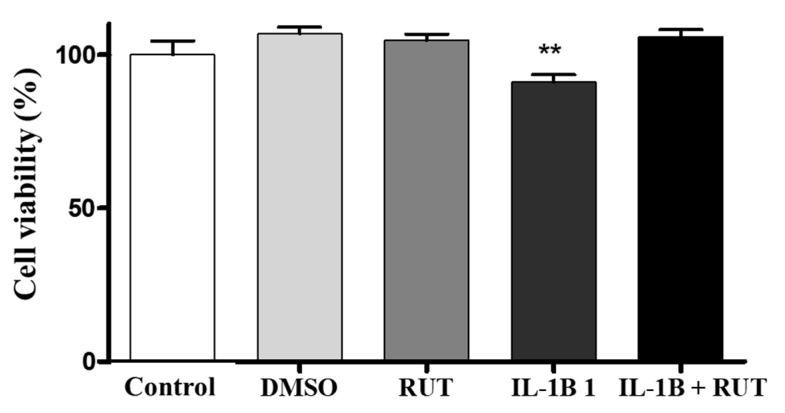
Analysis of cell viability by the MTT test on enteroglial cells. Enteroglial cells were exposed to IL−1β (5 ng mL^−1^) and untreated or treated with rutin (1 mmol) for 24 h. Cells under control conditions were treated with serum-free DMEM medium or 0.5% DMSO, a vehicle for drug dilution. Results expressed relative to control as 100% (** *p* < 0.01); *n* = 8 per group.

**Figure 9:**
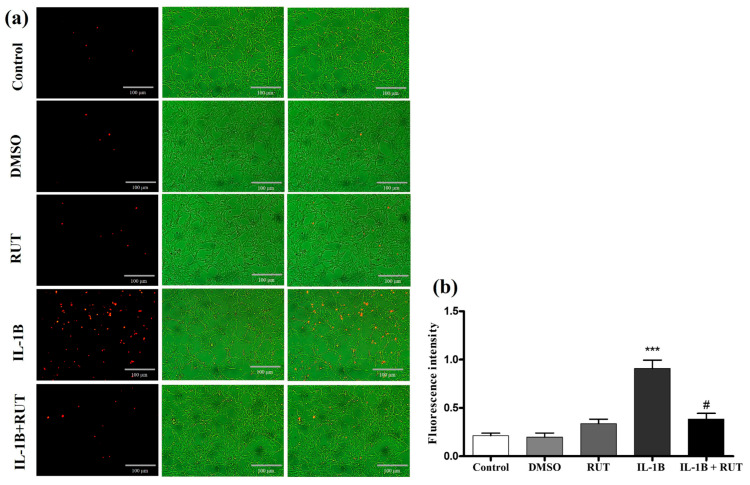
Propidium iodide test (IP). EGCs were exposed to IL-1β (5 ng mL^−1^) and untreated or treated with rutin (1 mmol) for 24 h. (**a**) Photomicrographs of cells stained with IP (control, DMSO, rutin, IL-1β, and IL-1β+ rutin). (**b**) Fluorescence intensity. *** *p* < 0.001 (IL-1β vs. DMSO) and # *p* < 0.05 (IL-1β+Rut vs. Rut). One-way ANOVA was conducted, followed by Bonferroni post-test; *n* = 8 per group.

In order to directly analyze the effect of rutin on EGCs, we performed the MTT test which establishes cell viability by measuring the functionality of mitochondrial dehydrogenases (Figure 8). After 24 h of exposure to IL-1β, a 15% reduction in the viability of these cells was observed (p < 0.01) when compared to the cultures under control conditions. However, the cultures of EGCs subjected to damage with IL-1β and treated with rutin (1 µg for 24 h) maintained a viability parameter similar to that of the control cultures. The same effect could be observed in the Propidium Iodide test (PI) (Figure 9). The EGC cultures subjected to IL-1β damage showed an increase in the intensity of fluorescence caused by IP (Figure 9a) (p < 0.001), which was not observed in the cell cultures that were stimulated with IL-1β and treated with rutin (Figure 9b) (p < 0.05).

## 3. Discussion

In this work, we analyzed the ability of the flavonoid rutin to modulate ENS in a PD model induced by 6-hydroxydopamine (6-OHDA). In rats, the unilateral intracerebral injection of 6-OHDA resulted in a selective degeneration of dopaminergic neurons, and this is an animal model that is widely used for the study of PD [20]. 6-OHDA induces a neurodegenerative process in the nigrostriatal system through the induction of oxidative stress, mitochondrial damage, inflammation, and abnormal protein aggregation, culminating in cell death. This model was well reproduced in the group of animals that received the nigrostriatal injection of the neurotoxin 6-OHDA, as demonstrated by the rotational test with apomorphine, where only injured rats were able to maintain a rotation above 100 turns in a period of 1 h. The behavioral tests corroborated this finding and allowed us to infer that animals injured with 6-OHDA, when treated with rutin, improve their behavioral parameters of exploration. Grooming involves an innate set of movements used by many mammalian species to care for the body [21,22,23]. Rodent studies have revealed that 6-hydroxydopamine lesions of dopaminergic neurons impair grooming, since dopamine is a crucial neurotransmitter for the implementation of the sequential grooming pattern [24]. Based on that, our results revealed a clear role in the reduced grooming observed in OHDA-treated animals with dopaminergic neuron degeneration. On the other hand, dopamine receptors are targets to modulate grooming actions in rats [23], and the stimulation of this dopamine receptor mediates the neuroprotection in a G2019S Lrrk2 genetic model of Parkinson’s disease [25]. Considering the role of the D2-dopamine receptor in the analgesic response of quercetin, an aglycone form of rutin [26], we can hypothesize a potential action of rutin in the D2-dopamine receptor as a mechanism underlying the reduced grooming in rutin-treated animals, which must be considered in our future studies.

The ENS has numerous similarities to the CNS and has been widely implicated in the pathophysiology of PD. Pathological changes in the ENS are involved in the gastrointestinal dysfunction that is often found in parkinsonian patients [27]. The flavonoid rutin has demonstrated neuroprotective and anti-neuroinflammatory effects in several in vitro models of neurodegenerative disorders [18,28]. Neuroinflammation is present in PD and has an important deleterious role in disease progression [29]. In light of this, one can infer that the flavonoid rutin may also have beneficial effects on the ENS. Some studies have already shown that flavonoids have a positive effect on the functioning of the intestinal barrier and that they protect intestinal cells from intestinal inflammation [17,24]. In this work, the flavonoid rutin was used as an alternative treatment suggestion for the damage caused by PD, observing its activity on the ENS. The results obtained showed that animals with Parkinsonism, when treated with the flavonoid rutin, showed a significant improvement in intestinal motility. It is known that intestinal dysfunctions are frequent and probable pre-motor manifestations of PD [30,31,32]. Therefore, substances that are capable of improving this condition should be considered in the treatment of PD.

In our study, we provided an assessment of the myenteric plexus dysfunction against the tissue reactivity of ileal and colonic segments. Furnnes [1] stated that the ENS of the small intestine and colon have complete reflex pathways that control intestinal motility. The integrity of the myenteric plexus is necessary for physiological intestinal motility, and PD produces intestinal damage that can compromise its integrity. Devos and colleagues [33] reported an increase in proinflammatory cytokines, such as TNF-α, IL-1β, IL-6, and IFN-γ, in the intestinal tissues of patients with PD, which may contribute to the inflammatory process. 

It is evidenced that the contractility of the smooth muscle segment of the colon (proximal and distal), but not in the ileum, was significantly increased after 6-OHDA induced central dopamine neurodegeneration four weeks after dopamine lesions [34,35]. In our data, we did not observe an increase in the contractility of the colonic or ileal segments in the 6-OHDA group after cholinergic receptor activation when compared to the control, which contrasts with previous data showing significantly increased contractions, particularly in the preparations of colonic segments [28,29]. However, treatment with rutin increased the carbachol reactivity of the ileum, but not the colon.

The ENS contains two types of muscle motor neurons: (i) enteric excitatory neurons that release acetylcholine (ACh) and tachykinins as transmitters, and (ii) enteric inhibitory neurons that have multiple transmitters, including a vasoactive intestinal peptide (VIP), a pituitary adenylyl cyclase-activating peptide (PACAP), and nitric oxide (NO) [1,2]. The primary transmitter of neurons appears to be NO [2], and deficits in transmission are observed when the enzyme neural nitric oxide synthase (nNOS) is knocked out [36]. Thus, NO is an important neurotransmitter for smooth muscle tissue relaxation and is involved in the modulation of intestinal motility [37,38].

In this study, we demonstrate that the relaxation induced by NPS (a NO donor) was significantly reduced in parkinsonian animals treated with rutin compared to the control group, suggesting that NO pathway signaling in the myenteric plexus seems to be impaired after rutin treatment favoring the contractility of the ileal segments. This suggests that there was a change in the chemical code of enteric neurons that resulted in a phenotypic shift that is necessary for the maintenance of cells expressing inhibitory neurotransmitters, as intestinal motility was not affected. Indeed, we demonstrated an increase in enteric neuron markers HuC/HuD in the ileum segments of animals with Parkinsonism. However, it is important to emphasize that HuC/HuD is a pan-neuronal marker, and therefore, we cannot assume that there was an increase in vipergic, nitrergic, or cholinergic neurons. However, the role of NO in the ENS is quite controversial [39]. Moreover, NO can be obtained via different pathways and stimuli. Martins-Perles et al., 2020 [40] demonstrated that the treatment with quercetin (flavonoid) increases NO bioavailability despite the reduction in the nitrergic subpopulation induced by diabetic neuropathy in the jejunum of rats.

Patients with PD demonstrate an increase in enteric glial reactivity through increased GFAP expression and reduced phosphorylation, which have been associated with degenerative CNS diseases [41]. Our results showed that there was an increase in immunoreactive S-100β enteric glial cells in both the 6-OHDA group and the 6-OHDA+rutin group. Similar to neuronal labeling, S-100β is a pan-glia, and therefore, the GFAP subpopulation is included in the total population. However, we cannot assume that there was an increase in GFAP expression. The experimental model used in this study may have influenced the functionality and response profile of the enteric glia in an inflammatory microenvironment in these gut regions, which could be investigated in future studies. In a study conducted by Christmann et al. (2021) [42] in a primary cell culture of the enteric glia of adult mice, it was also possible to observe that the flavonoid rutin can act as an antioxidant besides being able to neutralize the pathological impact caused by α-synuclein in the cells of the SNE in vitro.

## 4. Materials and Methods

### 4.1. Animals and Parkinson’s Disease Model

Thirty-two adult (3 months) male Wistar rats weighing 250 ± 20 g were obtained from the vivarium of the Institute of Health Sciences of the Federal University of Bahia and kept in an isolated room with access to water and food ad libitum under controlled conditions (20 ± 2 °C), relative humidity (45–55%), and 12:12 h light cycle. All animal research methods and procedures were approved by the Ethics Committee for the Use of Animals of the Institute of Health Sciences of the Federal University of Bahia (CEUA/UFBA) (Protocol CEUA No. 011/2017). The experiment was conducted in the form of blind, controlled, and randomized trials. The animals were divided into four groups: control (*n* = 8), 6-OHDA (*n* = 8), rutin group (*n* = 8), and 6-OHDA+rutin (*n* = 8) group. The number of animals in each group was determined using the hypothesis testing method, which relies on calculating the minimum number of experimental individuals required for the observed population differences to be statistically significant. To this end, G-Power software (version 3.0.10) and GraphPad Prism 5 software (version 5.01) were employed.

For the induction of PD, on day 1, a single dose of 21 μg of 6-hydroxydopamine (6-OHDA) (Sigma-Aldrich, St. Louis, MO, USA), diluted in 6 μL of 0.9% saline with 0.2% ascorbic acid, was injected in three different points into the right striatum (2 μg/μL of 6-OHDA in each point). The choice of this model of induction of Parkinsonism was based on De Araújo et al. (2023) [43]. For this, the rats were anesthetized with ketamine and xylazine (80 mg/kg; 10 mg/kg, i.p.) under stereotaxic conditions [44]. The injection was performed at the following coordinates: 2.5 mm mediolateral (ML) and 5 mm dorsoventral (DV) (first point); 3 mm ML, 0.5 mm anteroposterior (AP), and 6 mm DV (second point); and 3.7 mm mediolateral (ML), 0.9 mm anteroposterior (AP), and 6.5 mm dorsoventral (DV) (third point) [43]. In the control and rutin group, 2 μL of saline (0.9%) was injected in three different points into the right striatum. At the end of the injection, the needle was held in place for an additional 5 min to avoid backflow of the solution. Then, the wound was closed, and the animals were observed until fully recovered from the anesthesia. The animals (rutin group and 6-OHDA+rutin group) were submitted to oral treatment with the flavonoid rutin (10 mg/kg, orally) obtained from Sigma-Aldrich in 98% purity or 0.5% carboxymethylcellulose (CMC) vehicle for 14 days. The selected dose (10 mg/kg) and protocol of rutin administration were based on our previous studies that revealed the neuroprotective effect of oral doses of rutin in animochrome-induced dopaminergic degeneration in Wistar rat nigrostriatal system [43]. On the 13th day of the experiment, the whole gut transit test was performed, and the fecal output was evaluated. On the 14th day, the rats were submitted to the behavior tests. On the 15th day, the rats were anesthetized with ketamine and xylazine (80 mg/kg; 10 mg/kg, i.p.), underwent laparotomy to remove fragments of the intestine for the study of intestinal contractility and for the immunohistochemical analysis, and then euthanized by perfusion with phosphate-buffered saline (0.1 M, pH 7.4) (PBS 0.1 M). 

### 4.2. Open-Field Test

Locomotor activity and exploratory behavior were measured in an open-field apparatus. After 1 h of oral treatment with rutin (10 mg/kg) or vehicle, the animals were submitted to the test. Each animal was individually placed in the center of the apparatus and assessed for 6 min. The following measures were taken: locomotion (center and periphery), vertical counts (the number of times the animal stood on its hind legs), and grooming [45]. The arena was cleaned with alcohol (10%) after each session to avoid possible biasing of effects from olfactory cues. All animals were subjected to the same experimental conditions [46].

### 4.3. Cylinder Test

The cylinder test assesses the spontaneous forelimb lateralization, taking advantage of the natural exploratory instinct of rodents to a new environment [47]. Rats were placed individually inside a glass cylinder with mirrors located behind it to allow for a 360° vision for 5 min. No habituation of the rats to the cylinder was allowed before the test. Data were expressed as a percentage of contralateral touches, calculated as follows: Contralateral % = [ContraIpsi + Contra × 100].

### 4.4. Apomorphine-Induced Rotation Behavior

Rotational asymmetric behavior was assessed by blind observers. Apomorphine (3 mg/kg, i.p., Sigma Aldrich, St. Louis, MO, USA) was subcutaneously injected in the animals; after that, they were evaluated over 60 min as previously described [48]. The criterion for rotation was a 360° turn to the side contralateral to the injured hemisphere. To reduce stress, the rats were habituated for 1 h before the rotational test. Apomorphine-induced rotational behavior was assessed 14 days after the striatal injection. Control rats were also evaluated at the same time. Animals injured with 6-OHDA that did not present asymmetric rotational behavior were excluded. 

### 4.5. Whole Gut Transit Test and Fecal Output

On the 13th day of the experiment, the whole gut transit test was performed, and the fecal output was evaluated. Carmine, employed as a marker, was orally administered to each rat at 0.5 mL (3 g of carmine in 50 mL of 0.5% carboxymethylcellulose). Rats were then returned to individual boxes with white background, which were placed so as to distinguish stools colored by the marker from stools. The time taken for excretion of the head of the orally administered marker was measured. The endpoint was taken as the first appearance of one colored (red) pellet, and the appearance of the marker was based on visual observation. The observation was performed for 12 h after the administration of the marker. Fecal pellets’ wet and dry weights over the same time were also measured. Dry weight was determined after the pellets had been dried for 8 h in a laboratory oven at 80 °C [49]. 

### 4.6. Study of Intestinal Contractility 

The abdomen was opened, longitudinal strips of ileum and colon (1 cm) were quickly removed and transferred to Krebs solution (pH = 7.4), and the following composition was used (mmol/L): 128 NaCl, 4.5 KCl, 2.5 CaCl_2_, 1.18 MgSO_4_, 1.18 KH_2_PO_4_, 125 NaHCO_3_, and 5.55 glucose bubbled with 95% O_2_/5% CO_2_. The strips were mounted in an organ bath with a volume of 10 mL, containing Krebs solution, at 37 °C and pH = 7.4, oxygenated with a mixture of 5% CO_2_ and 95% O_2_. Intestinal contractility was recorded using a calibrated isometric force transducer (Insight, Ribeirão Preto, Sao Paulo, Brazil). Tension of 1 g was applied for an equilibrium period of 60 min with 4 washes every 15 min to remove metabolites. To assess tissue responsiveness, Carbachol (acetylcholine receptor agonist, 10^−10^ to 10^−4^ M), sodium nitroprusside (donor of nitric oxide, 10^−11^ to 10^−4^ M), and Krebs solution with KCl 80 mM were used. Following the methodology described, contractions of the muscle strips were recorded and analyzed [50,51].

### 4.7. Immunohistochemical Analysis

The ileum of each rat was fixed with 4% paraformaldehyde in 0.1 mol L^−1^ phosphate buffer, pH 7.4, for 8 h at 4 °C to evaluate the ENS. After fixation, segments were opened along the mesenteric edge and washed in PBS 0.1 M. Then, whole mounts of the myenteric plexus were prepared by microdissection. Whole mounts were then washed twice for 10 min in PBS solution containing 0.5% Triton X-100 (Sigma, Kawasaki, Japan). Then, they were incubated for 1 h in blocking solution (PBS + 0.5% Triton X–100 + 2% bovine serum albumin (BSA; Sigma) + 10% goat serum). After this period, the tissues were incubated for 48 h at room temperature in the blocking solution containing specific primary antibodies against HuC/HuD [52,53,54,55] (produced in mouse: 1:500; Molecular Probes, Eugene, OR, USA, cat. no. A21271) and S100β [52,56] (produced in rabbit; 1:200; Sigma, St. Louis, MO, USA, cat. no. S2644). Afterwards, whole mounts were washed three times in PBS solution + 0.5% Triton X-100 for five minutes and incubated for 2 h in room temperature with the secondary antibodies: Alexa Fluor 488−conjugated Donkey anti mouse IgG; 1:250 (Molecular Probes, Eugene, OR, USA, cat. no. A21202) and Alexa Fluor 568–conjugated Donkey anti-rabbit IgG; 1:500 (Molecular Probes, Eugene, OR, USA, cat. no. A10042). After this time, the whole mounts were washed again three times for five minutes in PBS solution and mounted on slides with Prolong^®^ Gold Antifade with DAPI (Molecular Probes) [54]. As a negative control, primary antibody was omitted [41,55]. Then, the tissue was observed and photographed under a fluorescence microscope (Olympus AX70, Olympus, Tokyo, Japan). Experiments were performed in triplicate. Quantification was analyzed using ImageJ 1.33u (Wayne Rasband, National Institute of Health, Bethesda, MD, USA).

### 4.8. Enteroglial Cell Culture (EGCs)

Rat-derived enteric glial cells [3] were cultured in a 100 mm diameter polystyrene plate (TTP, Trasadingen, Switzerland) in Dulbecco’s Eagle medium (DMEM) plus F-12 nutrient (DMEM-F1 (Gibco-Invitro2 medium, Grand Island, NY, USA) supplemented with 10% FBS (Fetal Bovine Serum), glutamine, 2 mM glucose acid, 3 mM, and penicillin (20 IU/mL) and streptomycin (20 µg/mL) (GIBCO, Grand Island, NY, USA). The cultivation was carried out in a biological greenhouse at 37 °C with 5% CO_2_ and 95% atmospheric air for approximately 3 days. As revised by Araújo et al. (2021) [14], the main event that regulates the secretion of IL-1β by the microglia/macrophages is the activation of inflammasome, a key function developed by the innate immune system in PD to sustain the neuroinflammatory process. Hence, cultures were exposed to IL-1β (5 ng/mL, Sigma Aldrich) directly added into the culture medium.

### 4.9. Evaluation of Cell Viability in EGC Cultures

Cytotoxicity was determined using the MTT test in EGC cultures. After the time of treatment, the medium was replaced by culture medium containing MTT at the final concentration of 1 µg/mL for 2 h, and cell viability was determined by the conversion of MTT to purple MTT formazan by the mitochondrial dehydrogenases of living cells [57]. Thereafter, cells were lysed with 50% (*v*/*v*) sodium dodecyl sulfate/dimethyl formamide (pH 4.7), and the plates were maintained at 37 °C overnight to dissolve the formazan crystals. The absorbance was analyzed using a spectrophotometer at 595 nm (Varioskan Flash, Thermo Fisher Scientific, Waltham, MA, USA). Three independent experiments were performed with 8 replicate wells for each analysis.

### 4.10. Propidium Iodide (PI) Incorporation Test in EGC Cultures

The test of Propidium Iodide (PI) incorporation in EGC cultures was performed to analyze cell viability. After 24 h exposure to IL-1β and/or rutin, or in control conditions, the culture medium was changed to a serum-free medium containing 5 μg/mL PI. The culture was incubated in a humidified atmosphere with 5% CO_2_ at 37 °C for 1 h. After the incubation time, the PI solution was discarded, and the EGCs culture was washed 3 times with PBS-glucose (0.6%). Following this, cells were observed using an Eclipse TS100 Fluorescence microscope (Nikon Instruments Inc., Melville, NY, USA). The red fluorescence intensity of the cortical area selected was analyzed with ImageJ 1.33u (Wayne Rasband, National Institute of Health, Bethesda, MD, USA). The data were evaluated as the ratio of fluorescence per area analyzed, and the fluorescence intensity was expressed by relative arbitrary densitometric units over the means of the control group.

### 4.11. Statistical Analysis

The data distribution was checked using D’Agostino–Pearson normality test. One-way ANOVA (analysis of variance) followed by the Student–Newman–Keuls test was used to determine the significant differences among groups differing in only one parameter. Student’s *t*-test was used to compare the two groups. Values of *P* lower than 0.05 were considered significant. All statistical analyses were performed using the software GraphPad Prism^®^ Version 5.01 (GraphPad Software Inc., La Jolla, CA, USA) or BioEstat 5.3^®^, with values of *p* < 0.05 considered as significant. The results are expressed as the mean ± SEM.

## 5. Conclusions 

In summary, our results indicate that the flavonoid rutin has modulatory activities on the ENS. The effects observed both in vivo and in vitro show that rutin has a protective effect against inflammatory damage without affecting the cell population, in addition to increasing the intestinal smooth muscle reactivity and improving the intestinal contractility in experimental models of PD, which may be related to the control of the NO signaling pathway, possibly via the soluble guanylyl cyclase receptor, the major NO cellular receptor for the cell that mediates a wide range of physiological effects through the elevation of intracellular cGMP levels. Overall, our results provide relevant answers about the effect of rutin on the ENS in an experimental model of PD, but we emphasize the need for further investigations in this area.

## Data Availability

Data are contained within the article.

## References

[1] Furness J.B. (2012). The enteric nervous system and neurogastroenterology. Nat. Rev. Gastroenterol. Hepatol..

[2] Furness J.B., Callaghan B.P., Rivera L.R., Cho H.-J. (2014). The Enteric Nervous System and Gastrointestinal Innervation: Integrated local and central control. Experimental Medicine and Biology.

[3] Rao M., Rastelli D., Dong L., Chiu S., Setlik W., Gershon M.D., Corfas G. (2017). Enteric Glia Regulate Gastrointestinal Motility but Are Not Required for Maintenance of the Epithelium in Mice. Gastroenterology.

[4] Gulbransen B.D., Sharkey K.A. (2012). Novel functional roles for enteric glia in the gastrointestinal tract. Nat. Rev. Gastroenterol. Hepatol..

[5] Spencer N.J., Hu H. (2020). Enteric nervous system: Sensory transduction, neural circuits and gastrointestinal motility. Nat. Rev. Gastroenterol. Hepatol..

[6] Gershon M.D., Rothman T.P. (1991). Enteric glia. Glia.

[7] Shannon K., Berghe P.V. (2018). The enteric nervous system in PD: Gateway, bystander victim, or source of solutions. Cell Tissue Res..

[8] Westfall S., Lomis N., Kahouli I., Dia S.Y., Singh S.P., Prakash S. (2017). Microbiome, probiotics and neurodegenerative diseases: Deciphering the gut brain axis. Cell. Mol. Life Sci..

[9] Herrero M.T., Morelli M. (2017). Multiple mechanisms of neurodegeneration and progression. Prog. Neurobiol..

[10] Schapira A.H.V., Chaudhuri K.R., Jenner P. (2017). Non-motor features of Parkinson disease. Nat. Rev. Neurosci..

[11] Schrag A., Horsfall L., Walters K., Noyce A., Petersen I. (2015). Prediagnostic presentations of Parkinson’s disease in primary care: A case-control study. Lancet Neurol..

[12] Filograna R., Beltramini M., Bubacco L., Bisaglia M. (2016). Anti-Oxidants in Parkinson’s Disease Therapy: A critical point of view. Curr. Neuropharmacol..

[13] Trist B.G., Hare D.J., Double K.L. (2019). Oxidative Stress in the Aging Substantia Nigra and the Etiology of Parkinson’s Disease. Aging Cell.

[14] De Araújo F.M., Cuenca-Bermejo L., Fernández-Villalba E., Costa S.L., Silva V.D.A., Herrero M.T. (2021). Role of Microgliosis and NLRP3 Inflammasome in Parkinson’s Disease Pathogenesis and Therapy. Cell. Mol. Neurobiol..

[15] Kujawska M., Jodynis-Liebert J. (2018). Polyphenols in Parkinson’s Disease: A systematic review of in vivo studies. Nutrients.

[16] De Araújo F.M., Ferreira R.S., Souza C.S., Dos Santos C.C., Rodrigues T.L.R.S., Silva J.H.C.E., Gasparotto J., Gelain D.P., El-Bachá R.S., Costa M.D.F.D. (2018). Aminochrome decreases NGF, GDNF and induces neuroinflammation in organotypic midbrain slice cultures. Neurotoxicology.

[17] Spagnuolo C., Moccia S., Russo G.L. (2018). Anti-inflammatory effects of flavonoids in neurodegenerative disorders. Eur. J. Med. Chem..

[18] Silva A.R., Pinheiro A.M., Souza C.S., Freitas S.R.V.-B., Vasconcellos V., Freire S.M., Velozo E.S., Tardy M., El-Bachá R.S., Costa M.F.D. (2007). The flavonoid rutin induces astrocyte and microglia activation and regulates TNF-alpha and NO release in primary glial cell cultures. Cell Biol. Toxicol..

[19] Khan M.M., Raza S.S., Javed H., Ahmad A., Khan A., Islam F., Safhi M.M., Islam F. (2011). Rutin Protects Dopaminergic Neurons from Oxidative Stress in an Animal Model of Parkinson’s Disease. Neurotox. Res..

[20] Ren R., Shi C., Cao J., Sun Y., Zhao X., Guo Y., Wang C., Lei H., Jiang H., Ablat N. (2016). Neuroprotective Effects of A Standardized Flavonoid Extract of Safflower Against Neurotoxin-Induced Cellular and Animal Models of Parkinson’s Disease. Sci. Rep..

[21] Berridge K.C. (1990). Comparative Fine Structure of Action: Rules of Form and Sequence in the Grooming Patterns of Six Rodent Species. Behaviour.

[22] Richmond G., Sachs B.D. (1980). Grooming in Norway Rats: The development and adult expression of a complex motor pattern. Behaviour.

[23] Young R.K., Thiessen D.D. (1991). Washing, drying, and anointing in adult humans (Homo sapiens): Commonalities with grooming sequences in rodents. J. Comp. Psychol..

[24] Berridge K.C. (1989). Substantia nigra 6-OHDA lesions mimic striatopallidal disruption of syntactic grooming chains: A neural systems analysis of sequence control. Psychobiology.

[25] Tozzi A., Tantucci M., Marchi S., Mazzocchetti P., Morari M., Pinton P., Mancini A., Calabresi P. (2018). Dopamine D2 receptor-mediated neuroprotection in a G2019S Lrrk2 genetic model of Parkinson’s disease. Cell Death Dis..

[26] Naidu P.S., Singh A., Kulkarni S.K. (2003). D2-dopamine receptor and alpha2-adrenoreceptor-mediated analgesic response of quercetin. Indian J. Exp. Biol..

[27] Lebouvier T., Chaumette T., Paillusson S., Duyckaerts C., Varannes S.B.D., Neunlist M., Derkinderen P. (2009). The second brain and Parkinson’s disease. Eur. J. Neurosci..

[28] Ferreira R.S., Teles-Souza J., Souza C.D.S., Pereira É.P.L., De Araðjo F.M., Da Silva A.B., Silva J.H.C.E., Nonose Y., Nðñez-Figueredo Y., de Assis A.M. (2021). Rutin improves glutamate uptake and inhibits glutamate excitotoxicity in rat brain slices. Mol. Biol. Rep..

[29] Rodríguez-Chinchilla T., Quiroga-Varela A., Molinet-Dronda F., Belloso-Iguerategui A., Merino-Galan L., Jimenez-Urbieta H., Gago B., Rodriguez-Oroz M.C. (2020). [18F]-DPA-714 PET as a specific in vivo marker of early microglial activation in a rat model of progressive dopaminergic degeneration. Eur. J. Nucl. Med. Mol. Imaging.

[30] Gil-Cardoso K., Ginés I., Pinent M., Ardévol A., Blay M., Terra X. (2016). Effects of flavonoids on intestinal inflammation, barrier integrity and changes in gut microbiota during diet-induced obesity. Nutr. Res. Rev..

[31] Dawson T.M., Dawson V.L. (2003). Molecular Pathways of Neurodegeneration in Parkinson’s Disease. Science.

[32] Das N.R., Sharma S.S. (2016). Cognitive Impairment Associated with Parkinson’s Disease: Role of mitochondria. Curr. Neuropharmacol..

[33] Devos D., Lebouvier T., Lardeux B., Biraud M., Rouaud T., Pouclet H., Coron E., Des Varannes S.B., Naveilhan P., Nguyen J.-M. (2013). Colonic inflammation in Parkinson’s disease. Neurobiol. Dis..

[34] Rodriguez-Pallares J., Parga J.A., Muñoz A., Rey P., Guerra M.J., Labandeira-Garcia J.L. (2007). Mechanism of 6-hydroxydopamine neurotoxicity: The role of nadph oxidase and microglial activation in 6-hydroxydopamine-induced degeneration of dopaminergic neurons. J. Neurochem..

[35] Murillo M.D.P., Johansson E., Bryntesson V., Aronsson P., Tobin G., Winder M., Carlsson T. (2023). 6-OHDA-Induced Changes in Colonic Segment Contractility in the Rat Model of Parkinson’s Disease. Gastroenterol. Res. Pract..

[36] Benvenuti L., D’antongiovanni V., Pellegrini C., Antonioli L., Bernardini N., Blandizzi C., Fornai M. (2020). Enteric Glia at the Crossroads between Intestinal Immune System and Epithelial Barrier: Implications for parkinson disease. Int. J. Mol. Sci..

[37] Rivera L.R., Poole D.P., Thacker M., Furness J.B. (2011). The involvement of nitric oxide synthase neurons in enteric neuropathies. Neurogastroenterol. Motil..

[38] Groneberg D., Voussen B., Friebe A. (2016). Integrative Control of Gastrointestinal Motility by Nitric Oxide. Curr. Med. Chem..

[39] Wiley J.W. (2007). The many faces of nitric oxide: Cytotoxic, cytoprotective or both. Neurogastroenterol. Motil..

[40] Martins-Perles J.V.C., Bossolani G.D.P., Zignani I., de Souza S.R.G., Frez F.C.V., de Souza Melo C.G., Barili E., Neto F.P.d.S., Guarnier F.A., Armani A.L.C. (2020). Quercetin increases bioavailability of nitric oxide in the jejunum of euglycemic and diabetic rats and induces neuronal plasticity in the myenteric plexus. Auton. Neurosci..

[41] Clairembault T., Kamphuis W., Leclair-Visonneau L., Rolli-Derkinderen M., Coron E., Neunlist M., Hol E.M., Derkinderen P. (2014). Enteric GFAP expression and phosphorylation in Parkinson’s disease. J. Neurochem..

[42] Christmann A., Gries M., Scholz P., Stahr P.L., Law J.K.Y., Schulte S., Martin M., Lilischkis R., Ingebrandt S., Keck C.M. (2021). The antioxidant Rutin counteracts the pathological impact of α-synuclein on the enteric nervous system in vitro. Biol. Chem..

[43] De Araújo F.M., Frota A.F., de Jesus L.B., Cuenca-Bermejo L., Ferreira K.M.S., Santos C.C., Soares E.N., Souza J.T., Sanches F.S., Costa A.C.S. (2023). Protective Effects of Flavonoid Rutin Against Aminochrome Neurotoxicity. Neurotox. Res..

[44] Paxinos G., Watson C. (2006). The Rat Brain in Stereotaxic Coordinates.

[45] Broadhurst P.L. (1959). Application of Biometrical Genetics to Behaviour in Rats. Nature.

[46] Archer J. (1973). Tests for emotionality in rats and mice: A review. Anim. Behav..

[47] Schallert T., Fleming S.M., Leasure J.L., Tillerson J.L., Bland S.T. (2000). CNS plasticity and assessment of forelimb sensorimotor outcome in unilateral rat models of stroke, cortical ablation, parkinsonism and spinal cord injury. Neuropharmacology.

[48] Kim Y.S., Joo W.S., Jin B.K., Cho Y.H., Baik H.H., Park C.W. (1998). Melatonin protects 6-OHDA-induced neuronal death of nigrostriatal dopaminergic system. Neuroreport.

[49] Devries M.P., Vessalo M., Galligan J.J. (2010). A deleção das subunidades dos receptores P2X2 e P2X3 não altera a motilidade do cólon de camundongos. Frente Neurosci..

[50] Farmer S.G., Laniyonu A.A. (1984). Effects of p-chlorophenylalanine on the sensitivity of rat intestine to agonists and on intestinal 5-hydroxytryptamine levels during Nippostrongylus brasiliensis infection. Br. J. Pharmacol..

[51] Frias B., Phillips A.A., Squair J.W., Lee A.H.X., Laher I., Krassioukov A.V. (2019). Reduced colonic smooth muscle cholinergic responsiveness is associated with impaired bowel motility after chronic experimental high-level spinal cord injury. Auton. Neurosci..

[52] Lin Z., Gao N., Hu H.-Z., Liu S., Gao C., Kim G., Ren J., Xia Y., Peck O.C., Wood J.D. (2002). Immunoreactivity of Hu proteins facilitates identification of myenteric neurones in guinea-pig small intestine. Neurogastroenterol. Motil..

[53] Schneider L.C.L., Nascimento J.C.P.D., Trevizan A.R., Góis M.B., Borges S.C., Beraldi E.J., Garcia J.L., Sant’Ana D.M.G., Buttow N.C. (2018). Toxoplasma gondii promotes changes in VIPergic submucosal neurons, mucosal intraepithelial lymphocytes, and goblet cells during acute infection in the ileum of rats. Neurogastroenterol. Motil..

[54] Trevizan A.R., Schneider L.C.L., Araújo E.J.d.A., Garcia J.L., Buttow N.C., Nogueira-Melo G.d.A., Sant’Ana D.d.M.G. (2019). Acute Toxoplasma gondii infection alters the number of neurons and the proportion of enteric glial cells in the duodenum in Wistar rats. Neurogastroenterol. Motil..

[55] Hermes-Uliana C., Panizzon C.P.D.N.B., Trevizan A.R., Sehaber C.C., Ramalho F.V., Martins H.A., Zanoni J.N. (2013). Is l-Glutathione More Effective Than l-Glutamine in Preventing Enteric Diabetic Neuropathy?. Dig. Dis. Sci..

[56] Ferri G.-L., Probert L., Cocchia D., Michetti F., Marangos P.J., Polak J.M. (1982). Evidence for the presence of S-100 protein in the glial component of the human enteric nervous system. Nature.

[57] Mosmann T. (1983). Rapid colorimetric assay for cellular growth and survival: Application to proliferation and cytotoxicity assays. J. Immunol. Methods.

